# Characterization of the plastid genome of *Cratoxylum* species (Hypericaceae) and new insights into phylogenetic relationships

**DOI:** 10.1038/s41598-022-23639-2

**Published:** 2022-11-05

**Authors:** Runglawan Sudmoon, Sanit Kaewdaungdee, Tawatchai Tanee, Pornnarong Siripiyasing, Unchaleeporn Ameamsri, Samsuddin Ahmad Syazwan, Shiou Yih Lee, Arunrat Chaveerach

**Affiliations:** 1 Faculty of Law, Khon Kaen University, Khon Kaen, Thailand; 2 Department of Biology, Faculty of Science, Khon Kaen University, Khon Kaen, Thailand; 3 Faculty of Environment and Resource Studies, Mahasarakham University, Maha Sarakham, Thailand; 4 Faculty of Science and Technology, Rajabhat Maha Sarakham University, Maha Sarakham, Thailand; 5 Mycology and Pathology Branch, Forest Biodiversity Division, Forest Research Institute Malaysia (FRIM), Kepong, Selangor Malaysia; 6 Faculty of Health and Life Sciences, INTI International University, Nilai, Negeri Sembilan Malaysia; 7 School of Life Sciences, Sun Yat-Sen University, Guangzhou, Guangdong China

**Keywords:** Biological techniques, Cell biology, Genetics, Molecular biology, Plant sciences

## Abstract

To expand the genomic information of Hypericaceae, particularly on *Cratoxylum*, we characterized seven novel complete plastid genomes (plastomes) of five *Cratoxylum* and two of its allied taxa, including *C. arborescens*, *C. formosum* subsp. *formosum*, *C. formosum* subsp. *pruniflorum*, *C. maingayi*, *C. sumatranum*, *Hypericum hookerianum*, and *Triadenum breviflorum*. For *Cratoxylum*, the plastomes ranged from 156,962 to 157,792 bp in length. Genomic structure and gene contents were observed in the five plastomes, and were comprised of 128–129 genes, which includes 83–84 protein-coding (CDS), 37 tRNA, and eight rRNA genes. The plastomes of *H. hookerianum* and *T. breviflorum* were 138,260 bp and 167,693 bp, respectively. A total of 110 and 127 genes included 72 and 82 CDS, 34 and 37 tRNA, as well as four and eight rRNA genes. The reconstruction of the phylogenetic trees using maximum likelihood (ML) and Bayesian inference (BI) trees based on the concatenated CDS and internal transcribed spacer (ITS) sequences that were analyzed separately have revealed the same topology structure at genus level; *Cratoxylum* is monophyletic. However, *C. formosum* subsp. *pruniflorum* was not clustered together with its origin, raising doubt that it should be treated as a distinct species, *C. pruniflorum* based on molecular evidence that was supported by morphological descriptions.

## Introduction

The family Hypericaceae Jussieu comprises nine genera and over 500 species worldwide. In general, members of Hypericaceae are further categorized into three different tribes viz. Cratoxyleae Bentham & J.D. Hooker, Hypericeae Choisy, and Vismieae Choicy^1^. As the smallest tribe in the family, two genera are recognized in *Cratoxyleae*, viz. *Cratoxylum* Blume and the monotypic genus, *Eliea* Cambess^2^. At present, there are seven accepted species of *Cratoxylum* Blume (Hypericaceae, Malpighiales), and three of them are recognized with at least two intraspecific identities^3^. Members of *Cratoxylum* are native to the tropical Asia region, widespread from India through South China to Malesia and are commonly soughed for their wood as a source of timber and charcoal production^4^. The great adaptability in harsh environments and fast-growing performance has warrant some of these species as potential replanting species that are useful for peatland rehabilitation strategies^5,6^.

Despite the potential as useful rehabilitation agents in peat swamp forests, genetic studies on *Cratoxylum* are limited. Genetic data of *Cratoxylum* are only restricted to short gene sequences derived from the plastid, mitochondrial and nuclear regions of either *C*. *arborescens* (Vahl) Blume or *C. cochinchinense* (Lour.) Blume as representative species of its genus in the reconstruction of the phylogenetic tree of Malpighiales^7–9^. The lack of the phylogenetic studies among species of *Cratoxylum* has hindered our understanding of this genus at its genetic level.

The plastid genome (plastome) is a valuable resource for molecular taxonomy research. Angiosperm plastomes are circular haploid genomes with a large single copy (LSC) region, two inverted repeats (IR), and a small single copy (SSC) region that are typically small in between 110–240 kbp in length^10^. Recently, researchers have shown great interest in obtaining the complete plastome sequences through next-generation sequencing technique. This is because when compared to short gene sequences, genome-scale datasets that are used in phylogenetics contain larger number of single nucleotide polymorphism, which could contribute to the reconstruction of a well-supported phylogenetic tree^11^. Owing to the advancement in sequencing technique and the availability of useful bioinformatic programs to aid in the assembly and annotation of the plastomes, to date, many complete plastome sequences have been made available publicly to decipher ambiguous phylogenetic relationships in complicated genera^12,13^. On the other hand, the nuclear DNA internal transcribed spacer (ITS) region has served to be useful in revealing the biparental inheritance of plants at a nuclear genome level^14^. Among all nuclear genes, amplification of the ITS sequence is known to be easier, and the genetic information provided is also useful to delimit individuals at an intraspecific level^15^.

Although records on published complete plastomes are increasing substantially over the years, genome data for Hypericaceae is still lacking. To date, published records on the complete plastome sequences of *Cratoxylum* are only limited to *C. cochinchinense*, in which at least three genome sequences of different accessions are available in the NCBI GenBank database (as of September 2021). In view of the need to expand the genomic information of Hypericaceae, we further sequenced and characterized the plastomes of five taxa of *Cratoxylum*, including *C. arborescens*, *C. formosum* subsp. *formosum* (Jack) Benth. & Hook.f. ex Dyer, *C. formosum* subsp. *pruniflorum* (Kurz) Gogelein, *C. maingayi* Dyer, and *C. sumatranum* (Jack) Blume, as well as two closely-related species, *Hypericum hookerianum* Wight & Arn. and *Triadenum breviflorum* Wall. ex Dyer. In order to reveal the phylogenetic relationship among species of *Cratoxylum*, we further performed phylogenetic analysis using the plastid protein-coding sequence (CDS) dataset and the nuclear DNA internal transcribed spacer (ITS) sequence region. The findings of this work will serve as important reference for the phylogenetic and evolutionary studies of Hypericaceae and Malpighiales.

## Results and discussion

### Plastome features

**Figure 1:**
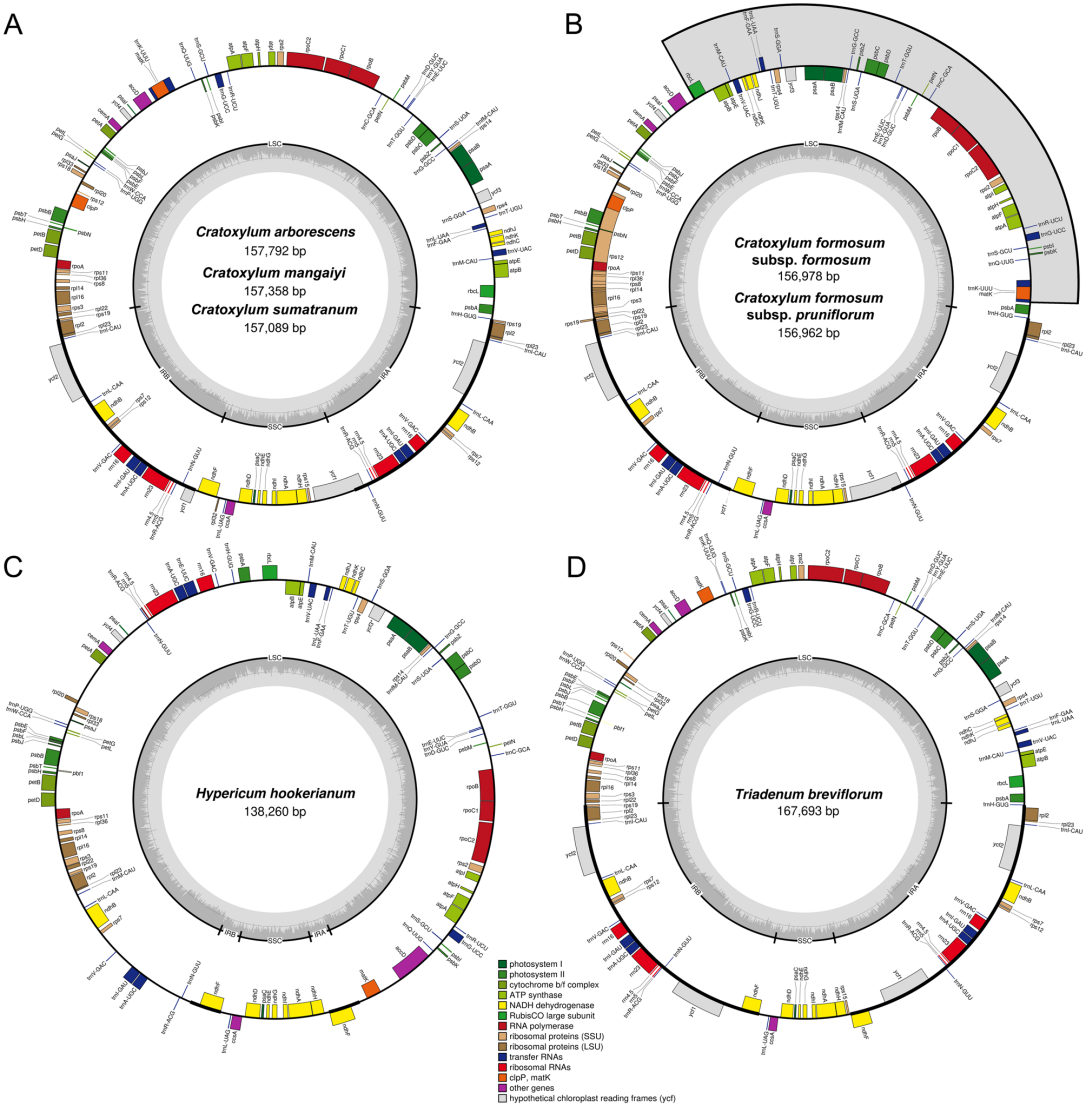
Genome structure and gene map of the seven taxa of Hypericaceae used in this study. *Cratoxylum arborescens*, *C. maingayi*, *C. sumatranum* (**A**); *Cratoxylum formosum* subsp. *formosum*, *C. formosum* subsp. *pruniflorum* (**B**); *Hypericum hookerianum* (**C**); *Triadenum breviflorum* (**D**). The inside circle genes are transcribed clockwise, and the outside circle genes are transcribed counter-clockwise. The color codes describe different functional groups of the genes. The thick lines indicate the boundary of the inverted repeats (IRA and IRB), demarcated between the large single-copy (LSC) and small single-copy (SSC) regions. The dark gray area in the inner circle represents genomic GC content, whereas light gray indicates AT content.

All seven plastomes obtained from this study exhibited a typical quadripartite structure, which comprised of a large single-copy (LSC) and a small single-copy (SSC) region that are separated by a pair of inverted repeats (IR) (Fig. 1). Plastome sizes were between 156,962 bp (*C. formosum* subsp. *pruniflorum* and 157,792 bp (*C. arborescens*) among the five taxa of *Cratoxylum*, while *H. hookerianum* and *T. breviflorum* were 138,260 bp and 167,693 bp in length, respectively (Table 1). A total of 128–129 genes were predicted in the plastome of the five taxa of *Cratoxylum*, which comprised of 83–84 CDS, 37 tRNA, and eight rRNA genes. *Cratoxylum formosum* subsp. *formosum* and *C. formosum* subsp. *pruniflorum* were short of one CDS compared to the other three species of *Cratoxylum*, which was the *rpl*32 gene that should be located at the SSC region (Table 2). There were 15 genes, including nine CDS and six tRNA genes, that contained one intron, while two genes, *clp*P and *ycf*3, contained two introns.

**Table 1:** General characteristics of complete plastid genomes of the seven taxa of Hypericaceae obtained in this study.

	Collector and collection number	Source of origin	Plastome features					GenBank accession number	
			CDS	tRNA	rRNA	Total length (bp)	GC content (%)	Plastome	ITS
*Cratoxulum arborescens*	Syazwan; SAS678	Selangor, Malaysia	84	37	8	157,792	36.1	MZ703418	MZ674200
*Cratoxylum formosum* subsp. *formosum*	A. Chaveerach; 1089	Udonthani, Thailand	83	37	8	156,978	36.3	MZ703419	MZ674201
*Cratoxylum formosum* subsp. *pruniflorum*	A. Chaveerach; 1090	Udonthani, Thailand	83	37	8	156,962	36.3	MZ703416	MZ674202
*Cratoxylum maingayi*	Syazwan; SAS679	Selangor, Malaysia	84	37	8	157,358	36.2	MZ703417	MZ674203
*Cratoxylum sumatranum*	A. Chaveerach; 1091	Udonthani, Thailand	84	37	8	157,089	36.3	MZ703415	MZ674204
*Hypericum hookerianum*	A. Chaveerach; 1092	Chiang Mai, Thailand	72	34	4	138,260	38.1	MZ714015	MZ703053
*Triadenum breviflorum*	Zhang et al.; TanCM704	Jiangxi, China	82	37	8	167,693	37.4	MZ714016	OM980718

**Table 2:** Genes predicted in complete plastid genome of the five taxa of *Cratoxylum* used in this study.

Category	Group of function	Genes
Self-replication related genes	Large subunit of ribosome proteins	*rpl*2(× 2)*, *rpl*14, *rpl*16*, *rpl*20, *rpl*22, *rpl*23(× 2), *rpl*32^a^, *rpl*33, *rpl*36
	Small subunit of ribosomal proteins	*rps*2, *rps*3, *rps*4, *rps*7(× 2)*, *rps*8, *rps*11, *rps*12, *rps*14, *rps*15, *rps*18, *rps*19(2)
	DNA-dependent RNA polymerase	*rpo*A, *rpo*B, *rpo*C1*, *rpo*C2
	rRNA genes	*rrn*4.5(× 2), *rrn*5(× 2), *rrn*16(× 2), *rrn*23(× 2)
	tRNA gene	*trn*A-UGC(× 2)*, *trn*C-GCA, *trn*D-GUC, *trn*E-UUC, *trn*F-GAA*, trnf*M-CAU, *trn*G-GCC, *trn*G-UCC*, *trn*H-GUG, *trn*I-CAU(× 2), *trn*I-GAU(× 2)*, *trn*K-UUU*, *trn*L-CAA(× 2), *trn*L-UAA*, *trn*L-UAG, *trn*M-CAU, *trn*N-GUU(× 2), *trn*P-UGG, *trn*Q-UUG, *trn*R-ACG(× 2), *trn*R-UCU, *trn*S-GCU, *trn*S-GGA, *trn*S-UGA, *trn*T-GGU, *trn*T-UGU, *trn*V-GAC(2), *trn*V-UAC*, *trn*W-CCA, *trn*Y-GUA
Photosynthesis related genes	Photosystem I	*psa*A, *psa*B, *psa*C, *psa*I, *psa*J
	Photosystem II	*psb*A, *psb*B, *psb*C, *psb*D, *psb*E, *psb*F, *psb*H, *psb*I, *psb*J, *psb*K, *psb*L, *psb*M, *psb*N, *psb*T, *psb*Z
	NADH oxidoreductase	*ndh*A*, *ndh*B(× 2)*, *ndh*C, *ndh*D, *ndh*E, *ndh*F(× 2), *ndh*G, *ndh*H, *ndh*I, *ndh*J, *ndh*K
	Cytochrome b6/f complex	*pet*A, *pet*B*, *pet*D*, *pet*G, *pet*L, *pet*N
	Cytochrome c synthesis	*ccs*A
	ATP synthase	*atp*A, *atp*B, *atp*E, *atp*F*, *atp*H, *atp*I
	Rubisco	*rbc*L
Other genes	Maturase	*mat*K
	Protease	*clp*P**
	Envelope membrane protein	*cem*A
	Subunit acetyl-CoA-carboxylase	*acc*D
Unknown function genes	Conserved hypothetical chloroplast reading frames	*ycf*1, *ycf*2(× 2), *ycf*3**, *ycf*4

Genes that contain duplicates are indicated in parenthesis. *Indicates gene containing single intron; **Indicates gene containing two introns; ^a^Indicates gene not found in *Cratoxylum formosum* subsp. *formosum* and *C. formosum* subsp. *pruniflorum.*

Although the gene content in plastome of *Cratoxylum* was consistent across the five taxa examined in this study, there was an inversed gene block arrangement detected in the LSC region, between *rbc*L and *trn*K-UUU genes (Fig. 1). The inversed gene block was approximately 55,000 bp in length, containing 28 CDS and 19 tRNA genes. By comparing to other plastomes of closely related families, we identified that the gene arrangement for the gene block in *C. cochinchinense*, *C. formosum* subsp. *formosum*, and *C. formosum* subsp. *pruniflorum* was similar to those of Bonnetiaceae, Calophyllaceae, Chrysobalanaceae, and Clusiaceae, i.e. *Bonnetia paniculata* (GenBank accession no. MK995182), *Caraipa heterocarpa* (GenBank accession no. MW853787), *Garcinia mangostana* (GenBank accession no. KX822787), and *Licania micrantha* (GenBank accession no. KX180080); while the gene arrangement for the gene block in *C. arborescens*, *C. maingayi*, and *C. sumatrana* was identical to those of Podostemaceae, i.e. *Marathrum capillaceum* (GenBank accession no. MN165813) and *Tristicha trifaria* (GenBank accession no. MK995179). This finding was congruent with a previous work, in which gene block inversion between *rbc*L and *acc*D was observed in two clusioid families, including Hypericaceae and Podostemaceae, as well as Papilionoideae^16^. For *H. hookerianum* and *T. breviflorum*, a total of 110 and 127 genes were predicted, including 72 and 82 CDS, 34 and 37 tRNA, as well as four and eight rRNA genes, respectively. The GC content of the plastome for the five taxa of *Cratoxylum* ranged between 36.1 and 36.3%, while GC content for the plastomes of *H. hookerianum* and *T. breviflorum* was 38.1% and 37.4%, respectively.

### Short and large sequence repeats

**Figure 2:**
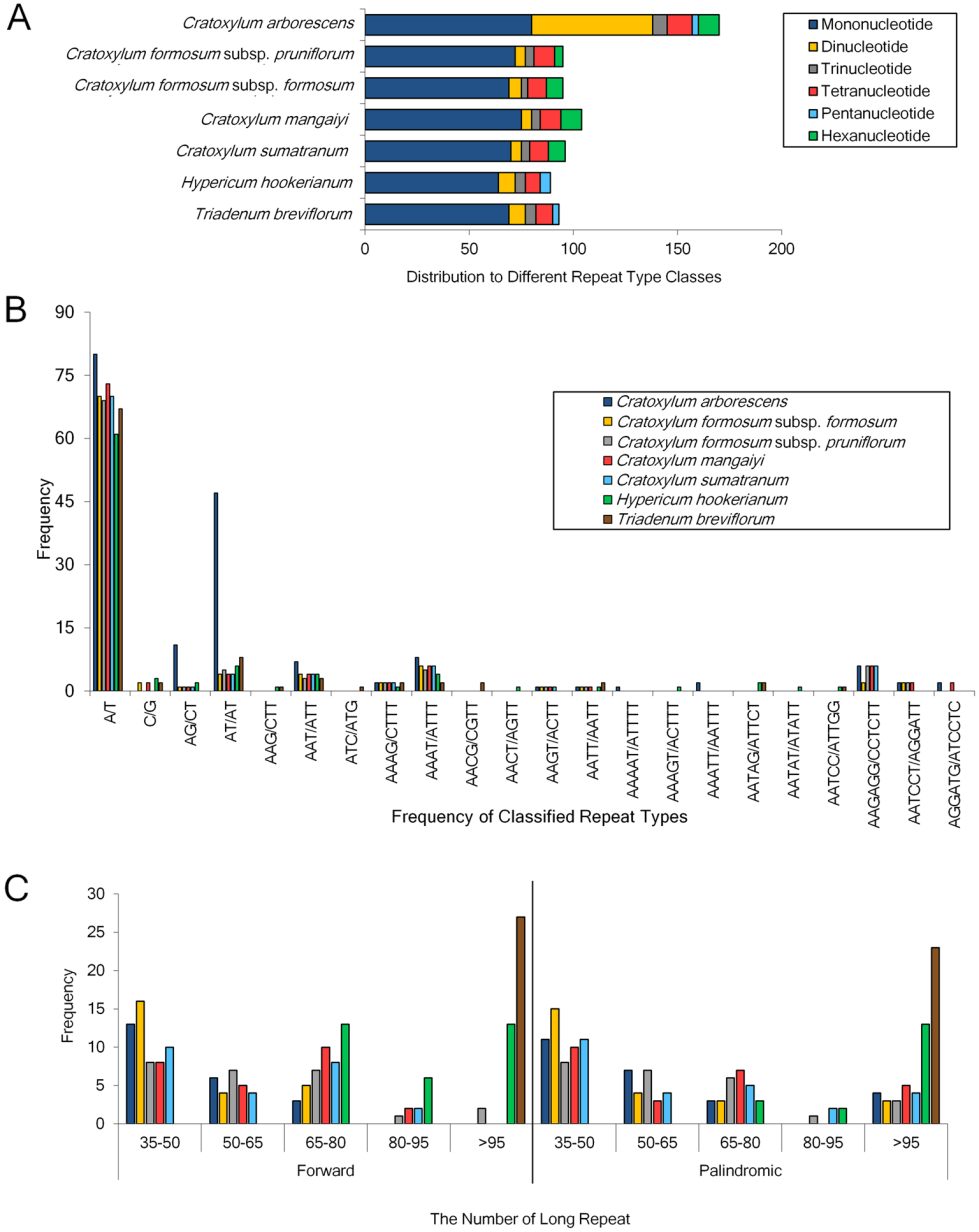
Repeat sequences in the plastid genomes of the seven taxa of Hypericaceae used in this study. Number of different simple sequence repeat types (**A**); the number of classified SSR repeat units (**B**); distribution and frequency of long repeat including forward and palindromic repeats (**C**).

Simple sequence repeats (SSRs) or microsatellites were short tandem repeats of 1–6 nucleotides and motifs at a specific locus are present in all genomes, particularly eukaryotes. Besides being developed as genome markers for the use in marker assisted selection, kinship, breeding, etc., SSRs contribute to the performance of important regulatory functions with the variations in their lengths at the coding regions^17,18^. In this study, the total SSRs detected in the plastomes of *C. arborescens*, *C. cochinchinense*, *C. formosum* subsp. *formosum*, *C. formosum* subsp. *pruniflorum*, *C. maingayi*, and *C. sumatranum* were 170, 103, 95, 95, 104, and 96, respectively (Fig. 2). The mononucleotide repeats were most abundant among all repeat types, ranging between 69 (*C. formosum* subsp. *formosum*) and 80 (*C. arborescens*); the frequency of mononucleotide repeat type A/T was greater than the repeat type C/G. It was worth noting that pentanucleotides were only found present in *C. arborescens*, including two AAATT/AATTT and one AAAAT/ATTTT repeat type. Large repeats were only recorded in forms of forward as well as palindromic repeats in the six plastomes assessed. All plastomes were identified with 25 each for both repeats, except for *C. sumatranum* that has 24 forward repeats and 26 palindromic repeats.

### Expansion and contraction of the IR regions

**Figure 3:**
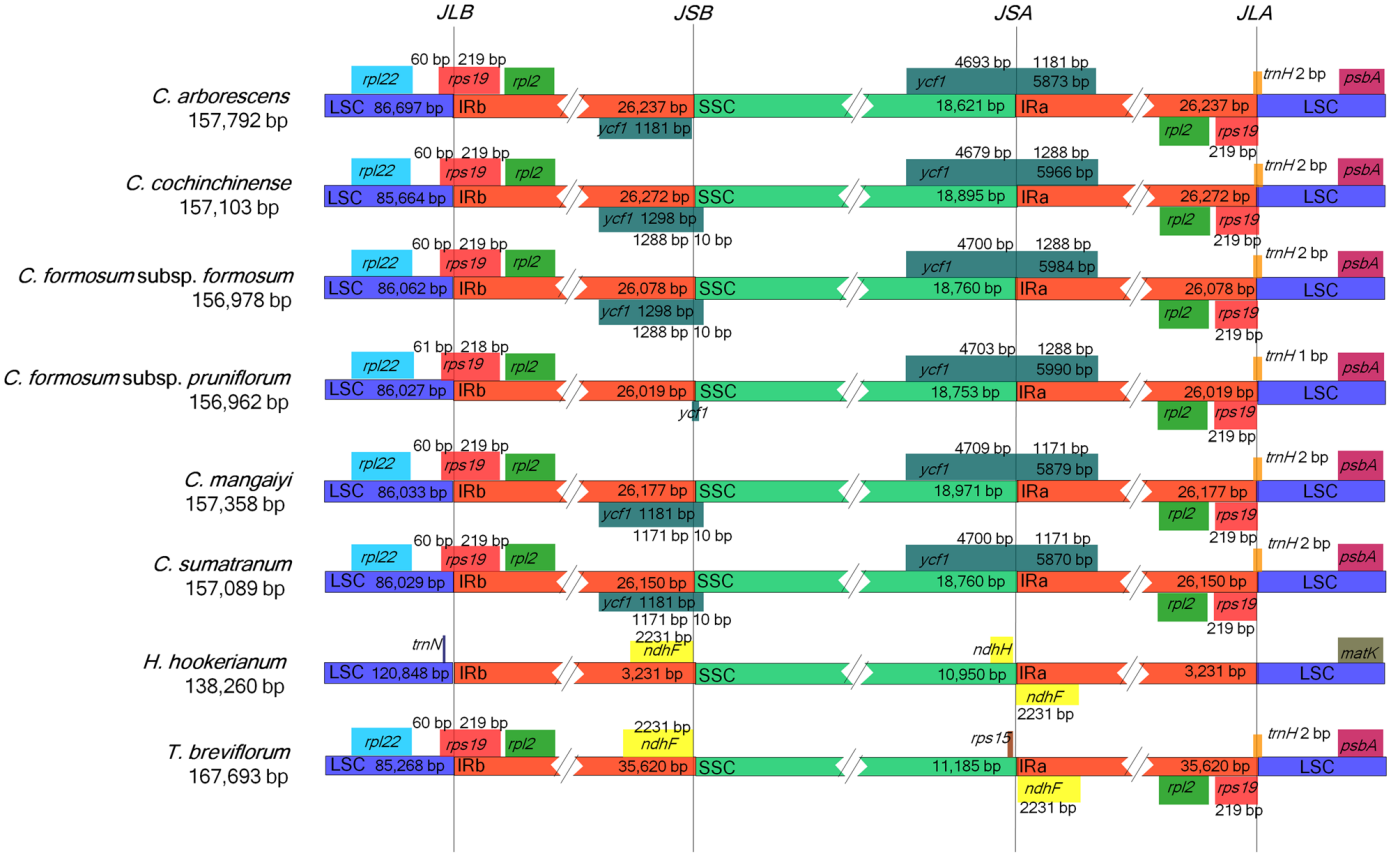
Inverted repeat (IR) border analysis based on the complete plastid genomes of eight taxa of Hypericaceae, including *C. arborescens*, *C. cochinchinense*, *C. formosum* subsp. *formosum*, *C. formosum* subsp. *pruniflorum*, *C. maingayi*, *C. sumatranum*, *Hypericum hookerianum*, and *Triadenum breviflorum*.

The genes adjacent to the IR junctions in the plastome of the six taxa of *Cratoxylum* examined displayed identical gene content (Fig. 3). The genes adjacent to the junction LSC/IRb (JLB) were *rpl*22 and *rps*19, with *rps*19 located across JLB. However, for the junction LSC/IRa (JLA), *rps*19 was intact in the IRa region, while *trn*H of the LSC region was recorded crossing over JLA. The *ycf*1 gene was placed across the junction SSC/IRa (JSA) for all six taxa of *Cratoxylum* examined, but for the junction SSC/IRb (JSB), only the *ycf*1 gene of four taxa of *Cratoxylum*, including *C. cochinchinense*, *C. formosum* subsp. *formosum*, *C. maingayi*, and *C. sumatranum*, were detected placing across JSB. The *ycf*1 of *C. arborescens* was still intact in the IRb region, while *ycf*1 gene of *C. formosum* subsp. *pruniflorum* was identified to be short in length and presumed to be a pseudogene, was located in the SSC region. When analyzed together with the six taxa of *Cratoxylum*, the gene content adjacent to the IR junctions in *H. hookerianum* and *T. breviflorum* exhibited some variations when compared to *Cratoxylum*. At JLA and JLB, the gene contents of *T. breviflorum* was similar to those of *Cratoxylum*, in which *rps*19 and *trn*H were placed across JLB and JLA, respectively. However, for *H. hookerianum*, the *trn*N gene was placed in the LSC region, next to JLB, while *rbc*L of the LSC region was the closest gene next to JLA. Both *H. hookerianum* and T*. breviflorum* had the *ndh*F genes located in the IR regions, adjacent to JSA and JSB, while at the SSC region, *ndh*A and *rps*15 were placed next to JSA in *H. hookerianum* and *T. breviflorum*, respectively.

### Comparative genomic analysis

**Figure 4:**
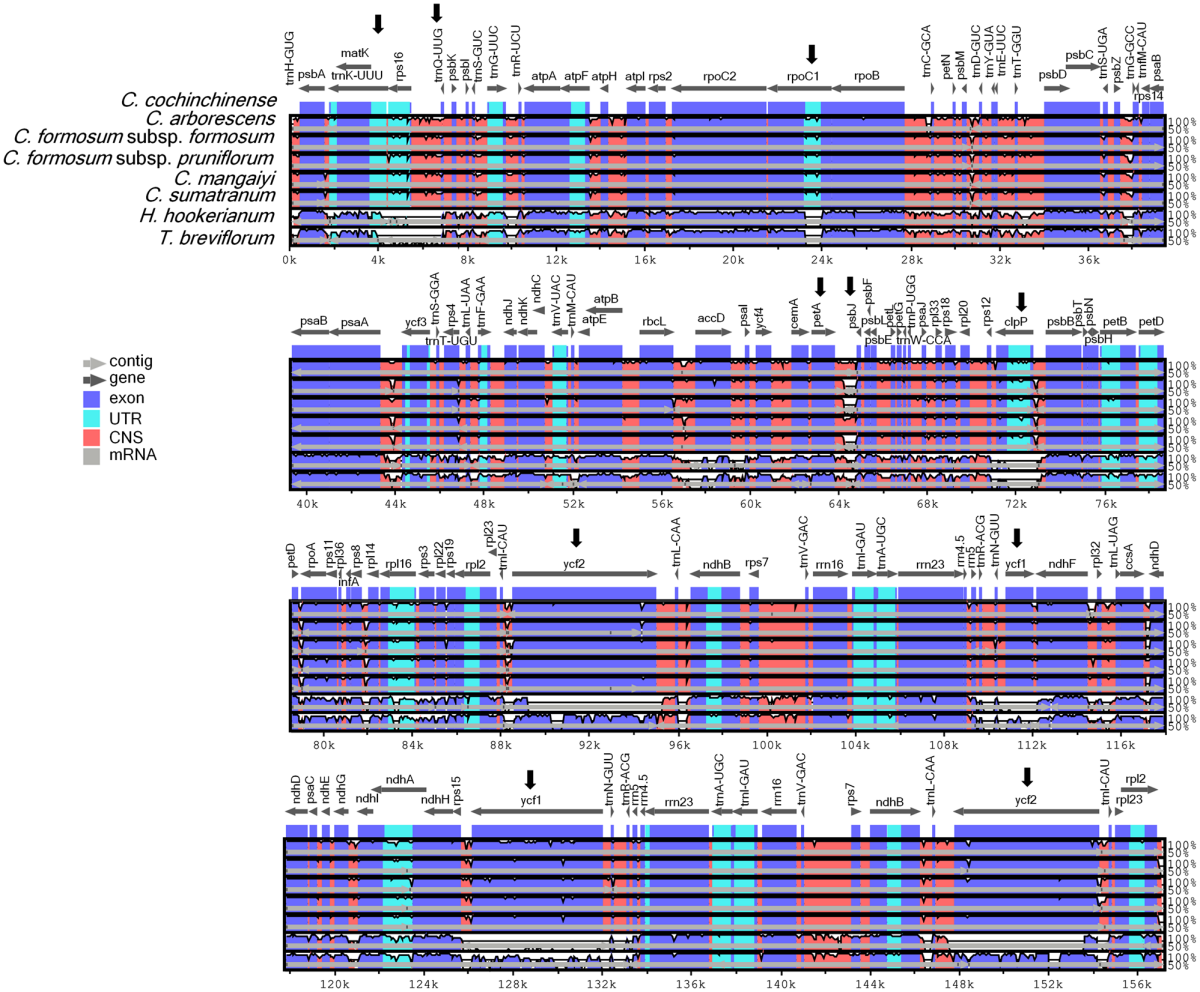
Genome comparative analysis of seven taxa of Hypericaceae used in this study, with the complete plastid genome sequence of *Cratoxylum cochinchinense* (MN399961) as the reference genome. Analysis was conducted using mVISTA under Shuffle-LAGAN mode. Figure legend describes the direction and types of gene regions using color codes. Probability threshold was set at 50%. Black arrows indicate regions that display distinct divergence in the plastid genome.

Genome comparison analysis of the complete plastome sequences revealed high conservatism across all taxa of *Cratoxylum*, with *C. cochinchinense* as the reference genome (Fig. 4). A small gap that resembles a variation in form of deletion, was observed at the intergenic spacer region *psb*J-*pet*A of *C. formosum* subp. *formosum*, *C. formosum* subsp. *pruniflorum*, and *C. sumatranum*. When compared to *H. hookerianum* and *T. breviflorum*, at least six large gaps could be observed across the plastome, indicating great variations in nucleotide sequence at genus level. These gaps were located at the *trn*Q-UUG-*trn*K-UUU, *clp*P, *trn*R-ACG-*ndh*F, *trn*B-ACG-*rps*15, and two *ycf*2 regions. The multiple sequence alignment of the six taxa of *Cratoxylum* was 169,031 bp in length, containing 1447 singletons and 22,090 parsimony informative sites. There were at least 1678 indel events identified, including a total of 23,364 indel sites.

### Phylogenetic inference

**Figure 5:**
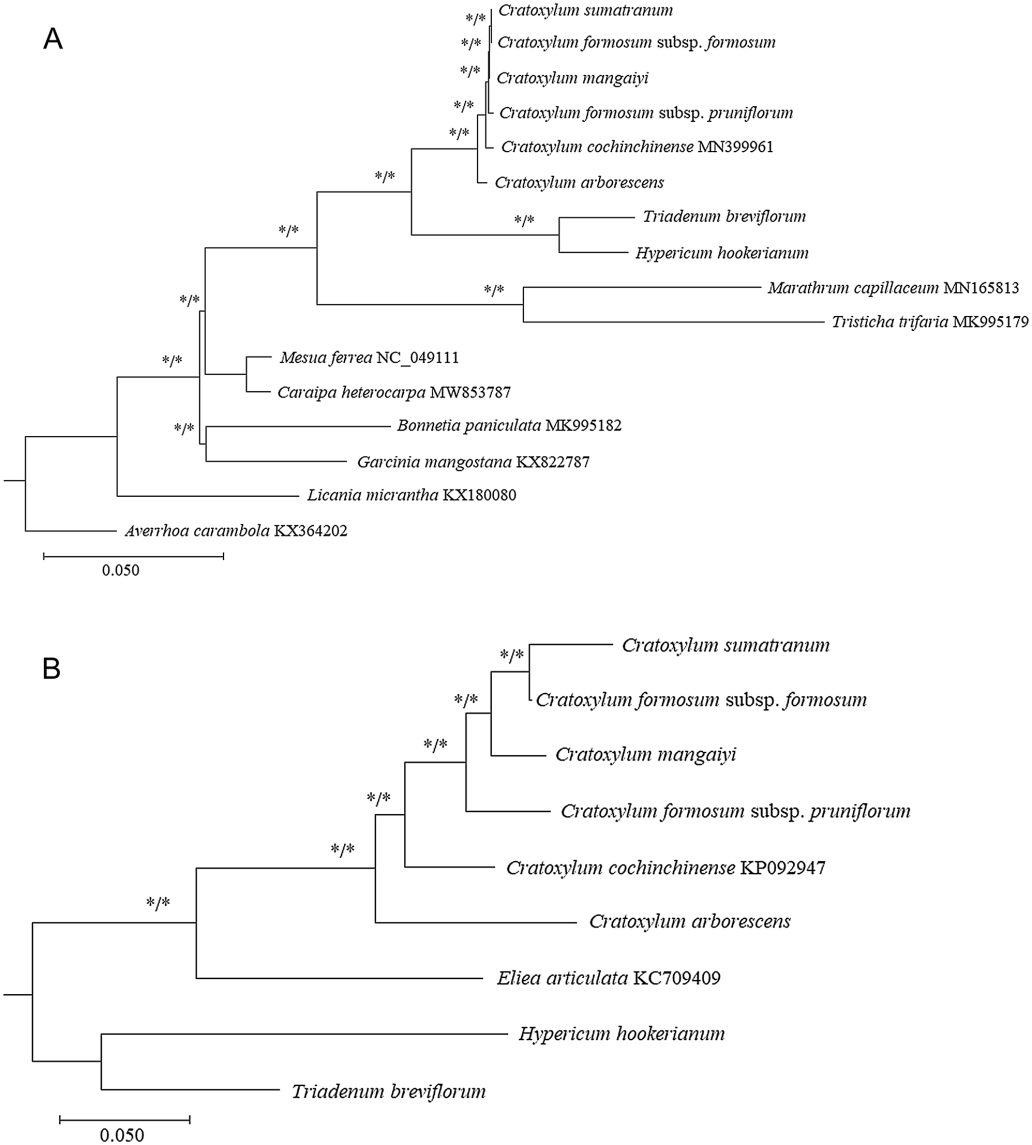
Phylogenetic trees of *Cratoxylum* and its allied taxa based on the concatenated protein-coding sequences derived from the plastid genome (**A**), as well as the nuclear DNA internal transcribed spacer (ITS) sequences (**B**). The phylogenetic tree was constructed using both maximum likelihood (ML) and Bayesian inference (BI). Bootstrap support (BS) and posterior probabilities (PP) that are considered reliable (BS: ≥ 75; PP: ≥ 0.95) are indicated with an asterisk (*).

The total length of the multiple sequence alignment of the concatenated CDS dataset was 89,82, while it was 807 bp before trimming and 723 bp after trimming for the ITS dataset. As both the ML and BI tree exhibited identical topology structure in both datasets, only the ML tree was presented in this study, with the BI posterior probability included at each of the branch nodes. In the CDS-tree, all branch nodes were well-supported (BS: ≥ 75; PP: ≥ 0.95); the phylogenetic relationship among all taxa included in the study was well-resolved (Fig. 5A). For *Cratoxylum*, all six taxa revealed a monophyletic relationship. *Cratoxylum arborescens* was recorded to diverge first from the other taxa, followed by *C. cochinchinense*, *C. formosum* subsp. *pruniflorum*, and *C. maingayi*. *Cratoxylum formosum* subsp. *formosum* was placed at the tip of the branch with *C. sumatranum*. For the ITS dataset, a similar tree topology was observed when compared to the tree reconstructed using the CDS-dataset (Fig. 5B); a monophyletic relationship was also observed in *Cratoxylum*, in which the molecular placement of all six taxa of *Cratoxylum* in the ITS-tree was identical to those presented in the CDS-tree. The phylogenetic relationship among all taxa of *Cratoxylum* used in this study was also well-resolved when using the ITS dataset (BS: ≥ 75; PP: ≥ 0.95).

### Conflicts on taxonomic identity of *C. formosum* subsp. *Pruniflorum*

It was noteworthy that *C. formosum* subsp. *pruniflorum* was not clustered together with its original, *C. formosum* subsp. *formosum* in the phylogenetic trees reconstructed using both the nuclear and plastid regions. Despite the ITS sequences, which are biparental inherited, could indicate possible hybridization in *C. formosum* subsp. *pruniflorum*; however, the well-resolved phylogenetic tree based on the maternal inherited plastid genes indicated that *C. formosum* subsp. *formosum* and *C. formosum* subsp. *pruniflorum* should be treated as two natural groups. Based on the literatures, *C. formosum* subsp. *pruniflorum* was first regarded as a distinct species, *Hypericum prunifolium* Wall^19^. However, the species has undergone several taxonomic revisions, before it was recognized as a subspecies to *C. formosum* in 1967^4^. Based on the description, the author emphasized that the key to differentiate between *C. formosum* subsp. *pruniflorum* and its original is based on the occurrence of an indumentum; both taxa exhibited high morphological similarities. Although it should not be a key morphological characteristic to differentiate the two taxa, *C. formosum* subsp. *pruniflorum* comes with pubescent sepals, while *C. formosum* subsp. *formosum* is glabrous at all parts, and they were geographically defined and hardly overlapping. Other morphological characteristics that were proposed to delimit *C. formosum* subsp. *pruniflorum* from *C. formosum* subsp. *formosum* were—the former has rusty, tomentose young twigs, pedicels and calyx, while the latter is glabrous; the former has short and truncated hypogynous scale, which is 0.7–0.8 mm long, while the latter has a linguiform hypogynous scale that is 2 mm long; the former has capsule that is ovoid-shaped and comes with 54–58 seeds, while the latter has ellipsoid-shaped capsule that is 36–46 seeded^20^.

To identify the genetic distance between *C. formosum* subsp. *pruniflorum* and its original based on the complete plastome and the ITS sequences, we conducted pairwise distance analysis on the complete plastome and ITS sequences and analyzed them separately. We found that the intraspecific pairwise distance was 0.00351, which was greater than the interspecific pairwise distance between *C. formosum* subsp. *pruniflorum* and *C. maingayi* (0.00159) at plastome level, while intraspecific pairwise distance was 0.0502, which was longer than the interspecific pairwise distance between *C. formosum* subp. *pruniflorum* and *C. maingayi* (0.0358) as well as *C. sumatranum* (0.0316) at the ITS level. There was no report on natural hybridization in *Cratoxylum*; despite that the pairwise distance was not a suitable parameter to tell closely related species apart, it was generally accepted that intraspecific pairwise distance of a species should be less than that of the interspecific pairwise distance under a regular basis^21,22^. On the other hand, the chromosome count in *C. cochinchinense* is n = 11^23^, while *C. formosum* subsp. *formosum* is known to be n = 7^24^. In general, the chromosome count in diploid plant species was often conserved intraspecifically under natural circumstances^25^. *Cratoxylum cochinchinense* was proposed to be conspecific to *C. formosum* at one time due to their identical morphological features, but the proposal was later denied; morphological variations between the two species were distinct in terms of their tree size, color of the bark, leaf structure, leaf shape, and staminal bundle of the flower^4^. Thus, we commented that cytology studies on *C. formosum* subsp. *pruniflorum* could provide useful insights to the genetic identity of the subspecies when compared to its original. Nevertheless, the finding between conventional taxonomic classification and molecular phylogenetic analysis in *Cratoxylum* was partly incongruent; the taxonomic identity of *C. formosum* subsp. *pruniflorum* to be accepted as a reduced taxon under *C. formosum* warrants further taxonomic revision on this genus. Based on the molecular evidence in this study, we believed that *C. formosum* subsp. *pruniflorum* should be considered as a natural group, and the species name *Cratoxylum pruniflorum* Kurz should be reinstated.

## Conclusion

This study has contributed to the expansion of genome data in Hypericaceae, with the characterization of novel plastomes of five taxa of *Cratoxylum*, as well as one each from *Hypericum* and *Triadenum*. The findings obtained from the well-resolved phylogenetic trees reconstructed using both the CDS and ITS datasets have provided insight to the molecular placement and evolution of *Cratoxylum*, in which the taxonomic identity of *C. formosum* subsp. *pruniflorum* to be recognized as a subspecies under *C. formosum* was questionable. Nonetheless, the molecular data obtained in this study will be a valuable resource for gaining a better understanding of Hypericaceae taxonomy and phylogeny.

## Materials and methods

### Plant materials

Fresh leaves of five taxa of *Cratoxylum* species, including *C. arborescens*, *C. formosum* subsp. *formosum*, *C. formosum* subsp. *pruniflorum*, *C. maingayi*, and *C. sumatranum*, as well as *Hypericum hookerianum* and *Triadenum breviflorum* (Supplementary Fig. 1) were collected from natural populations and ex-situ sites (Table 1). All the experiments were performed in accordance with relevant guidelines and regulations. The identities of each specimen were confirmed by the corresponding authors prior to specimen collection. Leaf specimens were kept in ziplock bags filled with silica gels and transported to respective local laboratories for total genomic DNA extraction.

### DNA extraction, genome sequencing and assembly

Total genomic DNA was conducted using DNeasy Plant Mini Kit (QIAGEN, Germany), based on the manufacturer’s protocol. The purity and quantity of the DNA extract were estimated using Qubit™ 4 Fluorometer (Thermo Fisher Scientific, USA). Next-generation sequencing was conducted on an Illumina NovaSeq platform (Illumina, USA) to obtain 350-bp paired-end reads. The NGS QC Toolkit v2.3 was used to trim off the adapter sequences^26^ and the plastome was assembled using NOVOPlasty v2.7.2^27^ with the *rbc*L gene of *C. cochinchinense* (GenBank accession no. MN399961) as the seed sequence. The assembled plastome was annotated and the inverted region junctions were identified using GeSeq v2.03^28^. The annotated plastome was manually checked for errors. The circular plastome map was visualized using OGDRAW v1.3.1^29^. All the plastome sequences obtained through this study were deposited into the NCBI GenBank database, under the accession number MZ703415—MZ703419, and MZ714015—MZ714016.

### Repeat analysis

In order to provide a better understanding between the plastomes of all species of *Cratoxylum* available online, the complete plastome sequence of *C. cochinchinense* (GenBank accession no. MN399961) was downloaded from the NCBI GenBank database. Subsequent genome comparative analyses were conducted with the inclusion of the genome data of this species. Using MISA-web, the SSRs of each plastome were identified^30^. The minimum number of repeat parameters were set for 10, 4, 4, 3, 3, and 3 for mono-, di-, tri-, tetra-, penta-, and hexanucleotide motifs, respectively. The large repeats, which includes the forward, palindromic, reverse, and complement repeats, were identified using REPuter^31^, in which the minimum repeat size was set at 30 bp and a Hamming distance of 3.

### Genome comparative and sequence divergence analyses

To detect the expansion and contraction of the IR region in the plastomes, the boundaries and junctions of the IR regions were visualized using IRscope program^32^ and further edited using Adobe Photoshop CS6 (Adobe, USA). Genome comparative analysis was carried out using mVISTA^33^ and genome alignment was performed under Shuffle-LAGAN mode. The plastome sequence of *C. cochinchinense* (GenBank accession no. MN399961) was selected as the reference genome. The number of polymorphic, parsimony informative, and indel sites present in the multiple genome alignment carried out using MAFFT v7^34^ were also calculated using DnaSP v5.10.01^35^.

### Polymerase chain reaction and Sanger sequencing

To obtain the ITS (ITS1-5.8S-ITS2) sequences of the five taxa of *Cratoxylum*, as well as *H. hookerianum*, and *T. breviflorum* used in this study, polymerase chain reaction (PCR) was carried out using a pair of universal primers, ITS5 5′-GGAAGTAAAAGTCGTAACAAGG-3′ and ITS4 5′-TCCTCCGCTTATTGATATGC-3′^36^. PCR amplification was conducted on a final reaction volume of 25 µL volume reaction containing 12.5 µL of the 2 × GoTaq^®^ Green Master Mix (Promega, USA), 10 µM of each forward and reverse primers, and 15 ng of DNA template. PCR amplification was programmed with thermal settings of an initial denaturation at 95 °C for 2 min, followed by 30 cycles of denaturation at 95 °C for 30 s, annealing at 55 °C for 30 s, extension at 72 °C for 1 min, and a final extension at 72 °C for 5 min. The amplicons were verified via gel electrophoresis and viewed under the UV machine prior to be sent for direct Sanger sequencing at both ends using an ABI 3730 DNA Analyzer (Applied Biosystems, USA). The resulting sequences were aligned and manually edited using MEGA7^37^ to obtain the clean sequences that will be subjected to phylogenetic analysis. The ITS sequence obtained from this study were deposited into the NCBI GenBank database under the accession numbers MZ674200—MZ674204, MZ703053, and OM980718.

### Phylogenetic reconstruction

The reconstruction of the CDS-based phylogenetic tree was conducted based on the concatenated CDS sequences of 14 taxa, in which eight are from Hypericaceae, while seven closely-related species, *Bonnetia paniculata* (Clusiaceae; GenBank accession no. MK995182), *Caraipa heterocarpa* (Calophyllaceae; GenBank accession no. MW853787), *Garcinia mangostana* (Clusiaceae; GenBank accession no. KX822787), *Licania micrantha* (Chrysobalanaceae; GenBank accession no. KX180080), *Marathrum capillaceum* (Podostemaceae; GenBank accession no. MN165813), *Mesua ferrea* (Calophyllaceae; GenBank accession no. NC_049111), as well as *Tristicha trifaria* (Podostemaceae; GenBank accession no. MK995179) that belong to Malpighiales were analyzed together. *Averrhoa carambola* (Oxalidaceae; GenBank accession no. KX364202) of Oxalidales was included as outgroup. Plastome sequences were aligned using MAFFT v7^34^ and phylogenetic analysis was conducted using both maximum likelihood (ML) and Bayesian inference (BI) method. For ML analysis, a generalized-time-reversible (GTR) model with gamma (+ G) (= GTR + G) was set and an ML tree was reconstructed using RAxML v8.2.11 under 1000 bootstrap replicates^38^. BI analysis was conducted using the MrBayes v3.2.7a^39^ pipeline available in the CIPRES Science Gateway^40^. A mixed substitution type and a 4 by 4 nucleotide substitution model were selected for the likelihood model, and a 2,000,000-generation Markov Chain Monte Carlo analysis and four Markov chains were implemented. Data sampling was conducted at every 100 generations, while the first 25% of trees was discarded as burn-in. The final tree results for both analyses were visualized using FigTree v.1.4.4^41^.

The ITS-based phylogenetic trees was reconstructed based on the ITS sequences of seven taxa from Cratoxyleae including the five taxa of *Cratoxylum* used in this study, *C. cochinchinense* (GenBank accession no. KP092947), and *Eliea articulata* (GenBank accession no. KC709409). *Hypericum hookerianum* and *T. breviflorum* were included as outgroups. Multiple sequence alignment was conducted using MUSCLE embedded in MEGA7^36^ and the alignment was trimmed using trimAL v1.2^42^ by selecting the gappyout option to reduce the systematic errors produced by poor alignment. ML analysis was conducted using MEGA7^37^, in which the “Find Best DNA/Protein Model (ML)” function embedded in MEGA7 calculated that the Tamura 3-parameter (T92) model with invariant sites included (+ I) (= T92 + I) was the optimal DNA substitution model. All sites were included in the analysis and calculation was conducted with 1000 bootstrap replicates. For BI analysis, calculations were performed using MrBayes v3.2.7a^39^ following the same parameters and settings as mentioned above.

## Supplementary Information

Supplementary Figure 1.

## Data Availability

The data that support the findings of this study are openly available in GenBank of NCBI at https://www.ncbi.nlm.nih.gov, accession number (MZ674200-MZ674204, MZ703053, MZ703415–MZ703419, MZ714015–MZ714016, and OM980718). The raw NGS data that support the findings of this study are available from the corresponding author, A.C., upon reasonable request.
