# Supplementary figures and images for: Characterization of the plastid genome of Cratoxylum species (Hypericaceae) and new insights into phylogenetic relationships
Source: Sci Rep. 2022 Nov 5;12:18810. doi: 10.1038/s41598-022-23639-2 (PMC9637187; doi:10.1038/s41598-022-23639-2)

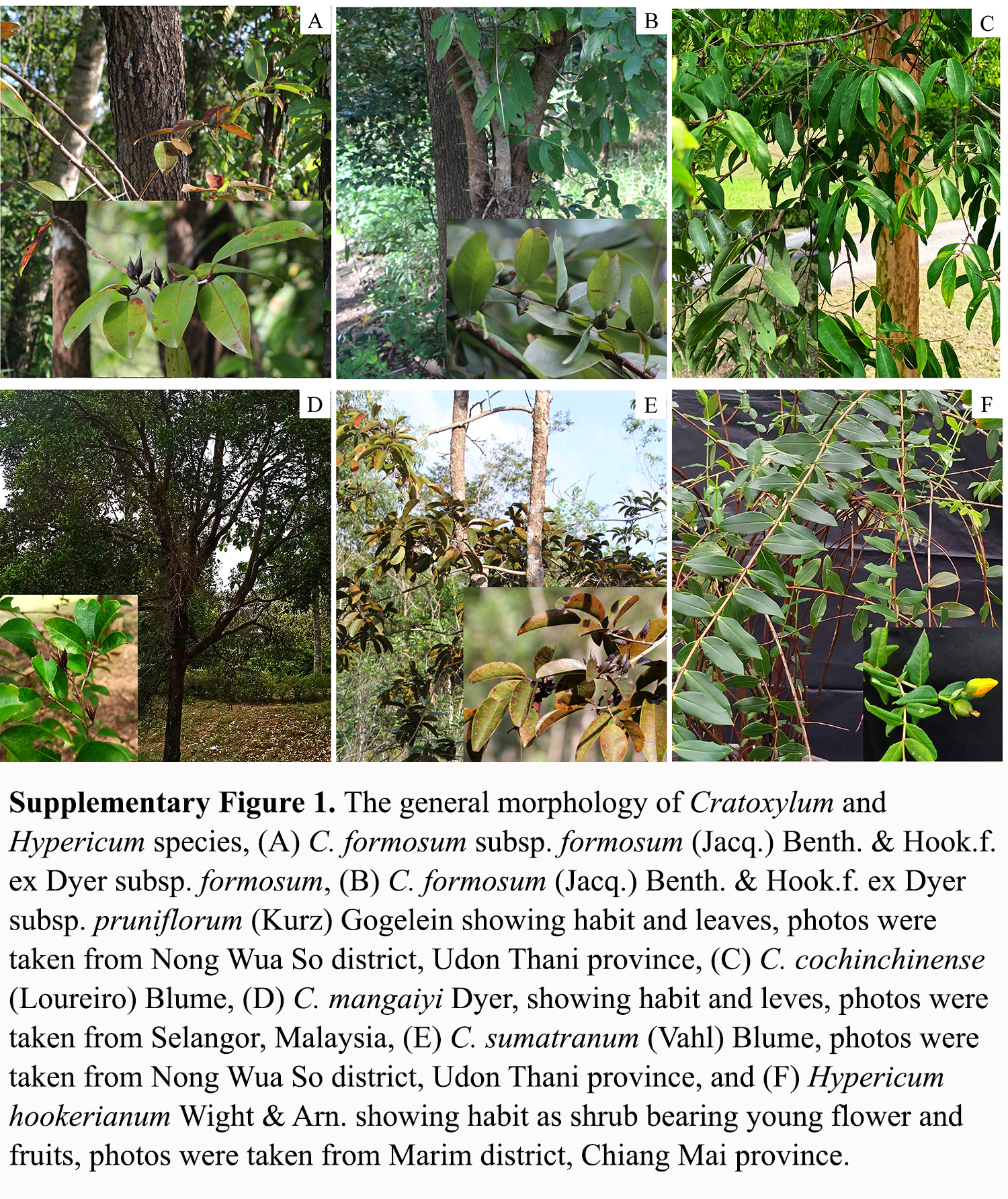

Supplement: Supplementary file 1 — Supplementary Figure 1. [file 41598_2022_23639_MOESM1_ESM.tif]
